# Adult-Onset Immunoglobulin A Vasculitis (IgAV) Presenting in a Middle-Aged Indian Male Patient

**DOI:** 10.7759/cureus.82735

**Published:** 2025-04-21

**Authors:** Abdalla Khalil, Naomi Oldham, Saju Beepaul, Elvin Chang

**Affiliations:** 1 Acute Medicine, Northwick Park Hospital London, London, GBR; 2 Dermatology, Northwick Park Hospital London, London, GBR; 3 Medical Imaging, Northwick Park Hospital London, London, GBR

**Keywords:** arthritis, cutaneous vasculitis of small vessels, immunoglobulin a nephropathy, immunoglobulin a vasculitis, palpable purpura

## Abstract

Immunoglobulin A vasculitis (IgAV) is a small vessel vasculitis that mainly affects the skin, gastrointestinal tract, and kidneys.

We present a case of a middle-aged male patient who presented to the emergency department with lower chest pain, colicky abdominal pain, and painful petechial rash. His inflammatory markers and IgA were raised with mild proteinuria.

Abdominal CT and endoscopy revealed gastrointestinal involvement, and immunofluorescence skin biopsy confirmed IgA deposition in the dermal vessels. He responded clinically to oral steroids, and his inflammatory markers trended down. After six days, he was discharged.

## Introduction

Immunoglobulin A vasculitis (IgAV), formerly known as Hench-Schonlein purpura, is a small vessel vasculitis characterized by the deposition of the dominant IgA1 immune complexes in the vessels of the target organs (predominantly capillaries, venules, and arterioles) [1].

Common clinical presentations include palpable purpura and petechiae (with a normal platelet count), arthralgia/arthritis, and gastrointestinal and renal involvement [2].

IgA vasculitis is the most common childhood primary vasculitis, but it is rare in adulthood [3]. The most common reported clinical presentation at onset in adults was non-thrombocytopenic purpura (97%), and the second most common presentation in adults was renal involvement (78%). Joint involvement was the third most common presentation (59%), followed by gastrointestinal involvement (39%) [3].

Many patients report infections preceding the onset of the symptoms of vasculitis (Streptococcus, parainfluenza, and human parvovirus B19), which are thought to trigger the disease. However, the full pathophysiology is not understood [4].

## Case presentation

A 57-year-old Indian male patient programmer with a past medical history of mild ankylosing spondylitis and suspected Raynaud’s disease presented to the Accident and Emergency (A&E) with a four-day history of sudden onset, sharp pleuritic left lower chest/subcostal pain.

He was found to be tachycardic at 121 beats/minute, with an ECG showing normal sinus rhythm and no ST changes. The rest of the blood tests were normal.

He was referred to an ambulatory clinic via the pulmonary embolism pathway, where CT Pulmonary angiography demonstrated no pulmonary embolism and normal lung parenchyma.

Three days later, he presented to A&E complaining of diffuse cramping abdominal pain, ongoing chest pain, and progressive painful and itchy bilateral rash on both legs, which started on his buttocks and spread over his legs and up to mid-back.

The patient did not report any recent fever, respiratory symptoms, cough, cold, or previous rashes. He has not traveled abroad recently and has had no contact with ill individuals.

**Figure 1:**
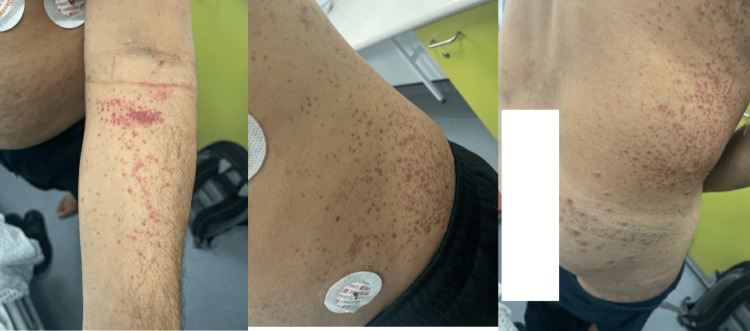
Rash type and distribution Widespread petechial rash at the arm, cubital area, buttock, flank, and back of the patient.

On examination, his heart rate was 110 beats/min, blood pressure was 126/70 mm Hg, temperature was 37 °C, respiratory rate was 20/min, and oxygen saturation was 97%. He had a widespread, tender, itchy petechial rash over the upper arms, back, abdomen, flanks, buttocks, and inner thighs (Figure 1).

His chest was clear on auscultation, and his heart examination was normal. Abdominal examination revealed generalized tenderness without guarding or rebound and normal bowel sounds. He had bilateral ankle mild pitting edema.

Blood tests on admission revealed high WBC, neutrophils, ESR, and CRP. Immunoglobulin A was raised too (Table 1).

**Table 1:** Patient's abnormal blood results on admission to the hospital. ALT: alanine aminotransferase; ALP: alkaline phosphatase; CRP: C-reactive protein; ESR: erythrocyte sedimentation rate; IgA: immunoglobulin A

Test	Result on admission	Normal level
White blood count	14.9 X10 ^9/L	3.6 - 11 X10^9/L
Hemoglobin	122 g/L	130 – 180 X10^9/L
Platelet count	542 X10^9/L	140 - 400 X10^9/L
Neutrophils	11.5 x10^9/L	1.8 - 7.5 X10^9/L
Sodium	136 mmo/L	135 – 145 mmol/L
Potassium	4.7 mmol/L	3.5 – 5.1 mmol/L
Creatinine	73 umol/L	66 – 110 umol/L
ALT	90 IU/L	5-40 IU/L
ALP	195 IU/L	30 -120 mg/dl
Bilirubin	14 umol/L	3-20 umol/L
Albumin	42 g/L	35-55 g/L
CRP	203 mg/dl	0 – 5 mg/dl
ESR	116 mm/hr	0-20 mm/hr
IgA	4.29 g/L	0.8 -3.0 g/L
Complement C3	1.63 g/L	0.75 – 1.75 g/L
Complement C4	0.36 g/L	0.20 - 0.4 g/L

An autoimmune screen was sent, including antineutrophil cytoplasmic antibodies (ANCA), antinuclear antibody (ANA), anti-double-stranded DNA (dsDNA), anti-RO, anti-Sm, complements, immunoglobulins, and rheumatoid factor, which were all negative apart from IgA.

His blood and urine cultures were negative, and his viral screen for COVID-19, flu, Epstein-Barr virus (EBV), cytomegalovirus (CMV), hepatitis B virus (HBV), hepatitis C virus (HCV), and HIV were all negative. His urine microscopy was normal, but the urine albumin creatinine ratio was mildly raised to 4.9 mg/mmol (<3 mg/mmol).

He was commenced on IV piperacillin/tazobactam to cover a possible abdominal infection. Following dermatology consultation, a provisional diagnosis of IgA vasculitis was made, and he was also commenced on oral prednisolone 30 mg once daily with a weaning course of 5 mg every five days.

His abdominal and chest pain improved, and his infection markers decreased. His ECG demonstrated normal sinus rhythm and no ST changes. A bedside ECHO demonstrated no pericardial effusion, normal systolic and diastolic function, and normal valves and pulmonary artery pressure.

**Figure 2:**
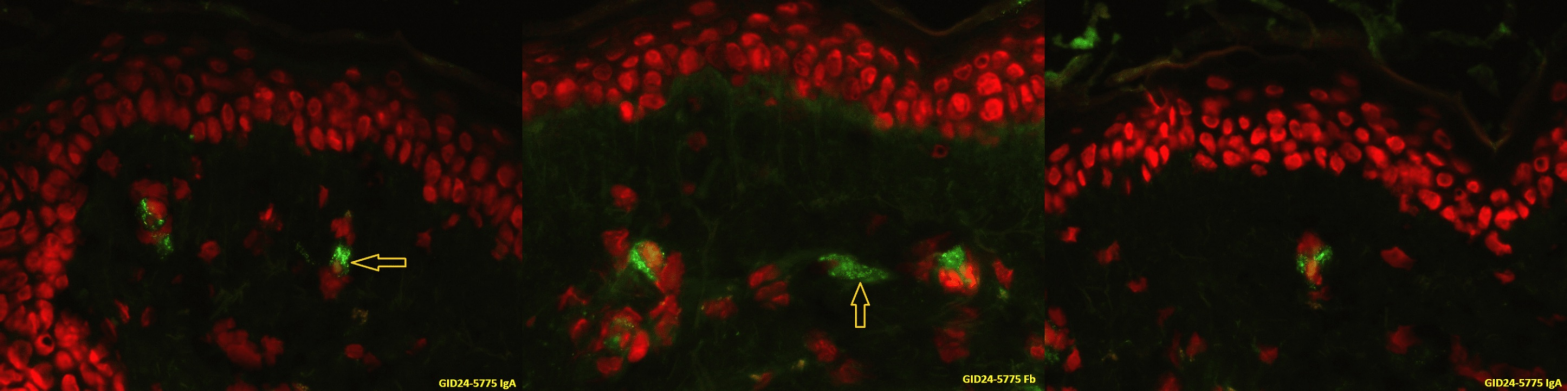
Immunofluorescence image of the skin biopsy. The IgA and fibrinogen are the granular fluorescent green colour in the dermal vessels (Yellow arrows)

A skin biopsy (from a recent rash on the back of the patient) was performed before his discharge, and immunofluorescence imaging showed IgA deposition in the subdermal vessels (Figure 2).

Oesophago-gastro duodenoscopy revealed erosive gastritis and duodenitis, which was felt likely secondary to vasculitis.

**Figure 3:**
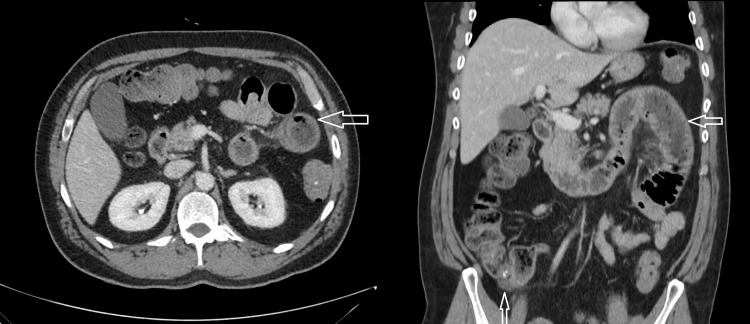
CT Abdomen and Pelvis with IV Contrast. Axial and Coronal view showing short segment of enteritis involving the proximal jejunum (left horizontal arrow). The focal area of fat stranding and small volume of free fluid surrounding the cecum suggests cecum inflammation/caecitis (right lower vertical arrow). Those areas of bowel inflammation may be secondary to the recent diagnosis of vasculitis.

CT abdomen and pelvis with intravenous contrast demonstrated a short segment of enteritis involving the proximal jejunum with a further focal area of fat stranding and a small volume of free fluid surrounding the cecum, suggestive of cecum inflammation. Multifocal areas of inflammation involving the small and large bowel were felt secondary to the recent diagnosis of vasculitis (Figure 3).

His abdominal and chest pain resolved, and his inflammatory markers were down-trending. He was discharged after six days with an outpatient follow-up with rheumatology, gastroenterology, and renal clinic.

## Discussion

The annual incidence of adult IgAV vasculitis varies among countries. It was reported at 6.21-20.4/100,000 in the UK, 6.1/100,000 in the Netherlands, 17.55/100,000 in southern Sweden, 12.9/100,000 in Taiwan, and 55.9/100,000 in Korea [3,5,6].

The disease is mostly mild and self-limiting when presenting in childhood, but it is more likely to follow a remission and relapsing course in adult IgA vasculitis [7,8].

In a French multicenter retrospective study of 260 adult patients with IgAV, 53% had gastrointestinal involvement. The most common initial presentation was abdominal pain 99%, intestinal bleeding was 31%, diarrhea was 26%, and surgical abdomen was 4%, and the most common finding at abdominal imaging was intestinal wall thickening 63%. The endoscopic abnormalities were 87%, mostly mucosal ulceration [9].

The European League Against Rheumatism/Pediatric Rheumatology International Trials Organization/Pediatric Rheumatology European Society (EULAR/PRINTO/PRES) published the classification criteria of IgA vasculitis in 2010. This was more sensitive and specific in the adult population than the old American College of Rheumatology criteria published in 1990. EULAR/PRINTO/PRES criteria for diagnosis of IgAV include purpura or petechiae and one of the following four criteria (abdominal pain, arthritis/arthralgia, renal involvement, and leukocytoclastic vasculitis with predominant IgA deposits or proliferative glomerulonephritis with predominant IgA deposits) [10].

In a recent retrospective study in Sweden of adults with IgAV from 2000 through 2019, the most common clinical presentation at onset was non-thrombocytopenic purpura (97%), renal involvement (78%), primarily mild, consisting of microscopic haematuria and non-nephrotic proteinuria. IgAV in adults frequently affects the kidneys and causes chronic kidney disease, but has a favorable outcome than other vasculitides affecting the kidneys [11].

Our patient presented with palpable petechial rash, abdominal pain, and gastrointestinal and renal involvement. His inflammatory markers and serum IgA were raised. His abdominal CT showed enteritis of the proximal part of the jejunum and inflammation of the caecum. His immunofluorescent image of the skin biopsy confirmed IgA deposition in the subdermal venules. 

Rarely, a patient with ankylosing spondylitis can develop leucocytoclastic vasculitis, which is another small vessel vasculitis, but no other organ is involved as in our patient.

## Conclusions

IgAV is a relatively rare disease in adults compared to children. Abdominal pain is one of the common presentations of IgAV which requires medical imaging and endoscopy to exclude other differential diagnoses and to assess the gastrointestinal involvement. Mild proteinuria may be the early renal involvement. Predominant IgA deposition in the dermal vessels is also a confirmatory finding.
